# Virulence typing and antibiotic susceptibility profiling of thermophilic *Campylobacter*s isolated from poultry, animal, and human species

**DOI:** 10.14202/vetworld.2018.1698-1705

**Published:** 2018-12-19

**Authors:** Neelam Rawat, Maansi, Deepak Kumar, A. K. Upadhyay

**Affiliations:** Department of Veterinary Public Health and Epidemiology, College of Veterinary and Animal Sciences, Govind Ballabh Pant University of Agriculture and Technology, Pantnagar, Uttarakhand, India

**Keywords:** antibiotic susceptibility, thermophilic *Campylobacters*, virulence

## Abstract

**Background and Aim:**

Campylobacteriosis finds its place among the four important global foodborne illnesses. The disease, though self-limiting, needs antibacterial therapy in extraintestinal complications. Therefore, the present study was designed to estimate the prevalence of thermophilic *Campylobacters* in poultry, animals, and humans of the Kumaon region of Uttarakhand.

**Materials and Methods:**

A total of 609 samples comprising of poultry ceca (n=116), poultry droppings (n=203), and feces of pigs (n=71), cattle (n=61), sheep (n=19), goat (n=17), human beings (n=88), and laboratory animals (n=34) (rats, rabbits, and guinea pigs) were collected. The thermophilic *Campylobacters*, *Campylobacter jejuni* and *Campylobacter coli* were confirmed using multiplex polymerase chain reaction. The isolates were also screened for the presence of virulence genes, and their antibiotic susceptibility testing was done against eight antibiotics.

**Results:**

An overall prevalence of 6.24% was revealed with highest from poultry ceca (15.52%), followed by poultry droppings (5.91%), cattle feces (4.92%), human stools (3.40%), and pig feces (2.82%). The virulence genes, namely *cadF*, *flaA*, *virB11*, and *pldA*, were present in 38 (100%), 37 (97.37%), 7 (18.42%), and 14 (36.84%) isolates, respectively. All the isolates were resistant to nalidixic acid, while all were sensitive to erythromycin and co-trimoxazole.

**Conclusion:**

It was concluded that the animals and humans in the region harbored the thermophilic *Campylobacters* which may contribute to the human illness. Resistance shown among the isolates may complicate the antimicrobial therapy.

## Introduction

Infections occurring due to the consumption of contaminated food are of a growing public health concern. These contaminated or unsafe foods pose a global threat affecting persons of all age groups. Food may get contaminated during any point of production and/or distribution by a number of microbes. Of all the food pathogens, *Campylobacters* have become a leading cause of enteric infections in both developing and developed countries. The *Campylobacters* have a broad animal reservoir. They are inhabitants of the intestinal tract of various domestic and wild animals, especially birds which are generally asymptomatic carriers. Therefore, inadequately cooked meat, particularly poultry, contaminated drinking water, unpasteurized milk, ready to eat food products, fecal runoff of birds and domestic animals contaminating surface water, and direct contact with animals act as the main source of the organism. Contaminated food is the primary mode of infection with poultry being the most common food source for humans [1]. Prevalence of *Campylobacters* in poultry has been reported by many authors [2-4]. In humans, the disease, campylobacteriosis is characterized by bloody diarrhea, fever, severe abdominal pain, and headache. Fever generally persists for up to 1 week in more than 90% of the patients; however, 50% of persons infected with *Campylobacters* remain asymptomatic [5]. It is a gastrointestinal disorder that generally affects infants, elderly people, and immunocompromised individuals. Most people suffering from campylobacteriosis recover within 2-5 days, but it may take up to 10 days in some cases. A very small dose of 500 cells may be infectious and sufficient to produce gastroenteritis. Common long-term complications of campylobacteriosis are Guillain-Barre syndrome, inflammatory bowel disease, bacteremia, rheumatoid arthritis, along with local complications such as cholecystitis, pancreatitis, peritonitis, massive gastrointestinal hemorrhage, thyroiditis, and prosthetic joint infection. Over 2.4 million persons annually or 0.8% of the total population [6] are affected making it a very important organism from a socioeconomic perspective. However, many of the cases go undiagnosed. The disease has a long back existence, and its recognition as a common infection is owed to improved laboratory methods. It still remains a high research priority to improve the strategies for management as well as prevention of the disease.

Molecular methods are useful in the identification of thermophilic *Campylobacters* as they enhance the sensitivity and specificity of the detection process. Polymerase chain reaction (PCR)-based species identification methods for *Campylobacter* spp. provide more reliable identification. Gorkiewicz *et al*. [7] recommended 16S*r*RNA sequence analysis as an effective, reliable, and rapid procedure for the specific identification of *Campylobacters*. Molecular techniques have come a long way in the characterization of microbes. Despite higher recovery rates of *Campylobacters* as foodborne pathogens, the specific virulence and pathogenic mechanisms of *Campylobacter* spp. infection is still poorly understood. The putative virulence factor for adhesion (*cad*F, *dna*J, *jlp*A, *pld*A, *rac*R, and *vir*B11) and invasion of epithelial cells (*iam*A, *cia*B, and *ceu*E), toxin production (*cdt*A, *cdt*B, *cdt*C, and *wla*N), and flagellar motility (*fla*A, *fla*B, *flh*A, *flh*B, *flg*B, *flg*E2, *fli*M, and *fli*Y) are thought to be important virulence mechanisms. Different studies in this regard have indicated the role of different virulence markers for adherence, invasion, and colonization of the organism in humans and animals. These virulence-related factors contribute to survival and establishment of the disease in host, thus modulating the clinical presentation of the disease. Antimicrobial agents are used for the early recovery of extraintestinal infections in immunocompromised patients or whenever clinical therapy is needed. The emergence of antimicrobial-resistant (AMR) *Campylobacters* has alerted toward the chances of increased invasive illness. The resistance has been linked to the illicit use of antimicrobials in animal feeds, food animals, and flock treatment of animals. Few antibiotics such as macrolides (erythromycin [ERY]) and fluoroquinolones (ciprofloxacin [CIP]) used as drug of choice have shown resistance against *Campylobacter* spp. [8]. Newer antibiotics are being continuously added to the list every year.

The present study was, therefore, undertaken to characterize the isolated *Campylobacters* on the presence of virulence genes and the phenotypic antibiotic resistance.

## Materials and Methods

### Ethical approval

Samples were collected as per standard sample collection procedure.

### Sample collection and processing

A total of 609 samples comprising of poultry ceca (n=116), poultry droppings (n=203), and feces of pigs (n=71), cattle (n=61), sheep (n=19), goat (n=17), human beings (n=88), and laboratory animals (n=34) (rats, rabbits, and guinea pigs) were collected from Uttarakhand state of India. Sterile 100 ml Whirl-Pak bags (Nasco, Fort Atkinson, WI) were used to collect the samples. The samples were collected aseptically and immediately brought to the laboratory for processing as per previously published protocols [9,10].

In brief, the poultry ceca and poultry fecal samples were streaked directly onto the modified charcoal-cefoperazone-deoxycholate agar (mCCDA, HiMedia, India) plates and incubated at 42°C with 5% CO_2_ in a CO_2_ incubator for 48 h [10]. However, human and other animal fecal samples (1 g) were initially enriched in 9 ml Bolton Broth (Oxoid, UK) supplemented with 5% sheep blood. Thereafter, a loopful of the enriched broth suspension was streaked onto mCCDA plates and was incubated at the same time-temperature combination. The characteristic *Campylobacter* colonies (1-2 mm size, circular, flat to slightly raised, sticky, spreading, and shiny gray) were selected from each plate and tested biochemically.

### Biochemical and molecular confirmation

All the presumptive Campylobacter isolates showing catalase and oxidase positive reaction while urease and TSI negative reaction were subjected to DNA isolation using Hi-PurA genomic DNA extraction kit (Hi-media).

A simplex PCR assay targeting the 16*SrRNA* [11] was used for the *Campylobacter* genus identification. The primer sequence and the cyclic conditions were used as per Linton *et al*. [11] for *Campylobacte*r genus. All PCR confirmed *Campylobacter* isolates were stored as 20% glycerol stock at −80°C.

### Multiplex PCR

Multiplex PCR was carried out for the identification of genus as well as species of the isolates. Multiplex PCR included amplification of *cad*F gene for genus identification [12], whereas *hip*O gene [11] and *asp* gene [11] amplification for the identification of *Campylobacter jejuni* and *Campylobacter coli*, respectively. The primer sequence and the cyclic conditions were used as per Linton *et al*. [11] and Nayak *et al*. [12] for *Campylobacte*r genus and species, respectively.

### Detection of virulence genes

All *Campylobacter* isolates were screened for the presence of various virulence genes by PCR. Virulence genes screened were *fla*A [13], *vir*B11 [14], and *pld*A [15]. PCR reaction and cycling conditions were used as described earlier in respective references.

### Antimicrobial susceptibility testing

The AMR profile of *Campylobacter* isolates was determined using standard Kirby-Bauer disc diffusion method as described by Taremi *et al*. [16]. A total of 38 revived isolates were tested against a panel of eight antibiotics that included ampicillin (AMP, 10 µg), gentamicin (GEN, 10 µg), ERY (15 µg), levofloxacin (LE, 5 µg), CIP (5 µg), nalidixic acid (NA, 30 µg), ceftriaxone (CTR, 30 µg), and co-trimoxazole (COT, 25 µg) (HiMedia). The isolates were revived on mCCDA plates supplemented with FD009 supplement. The growth suspension prepared in Tryptic soy broth and compared with 0.5 McFarland standard was spread on Mueller-Hinton agar plates supplemented with 7% sheep blood and incubated at 42°C in a CO_2_ incubator at 5% CO_2_ tension for 24 h. Zone diameter was measured and breakpoints were interpreted based on the recommendations of the Clinical and Laboratory Standards Institute standards for disc diffusion assay (CLSI 2016).

## Results

### Isolation and molecular confirmation

Of 609 samples (poultry ceca [n=116], poultry droppings [n=203], and feces of pigs [n=71], cattle [n=61], sheep [n=19], goat [n=17], human beings [n=88], and laboratory animals [n=34]) screened, 38 were confirmed as positive for *Campylobacter* yielding a prevalence of 6.24%.

**Figure-1:**
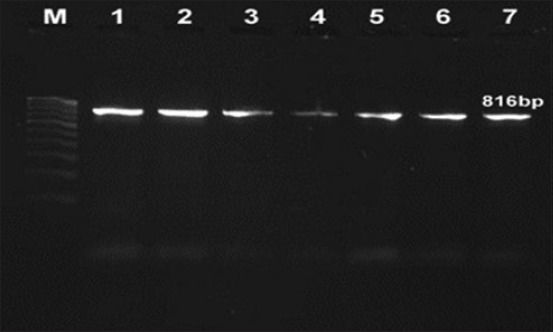
Agarose gel showing polymerase chain reaction product specific for genus *Campylobacter* spp. (16*SrRNA*), Lane M: 100bp ladder, Lanes 1-7: *Campylobacter* isolates (816 bp).

All the isolates produced a genus-specific amplicon of 816 bp in 16*SrRNA* *Campylobacter* genus-specific PCR (Figure-1).

### Multiplex PCR for species identification

**Figure-2:**
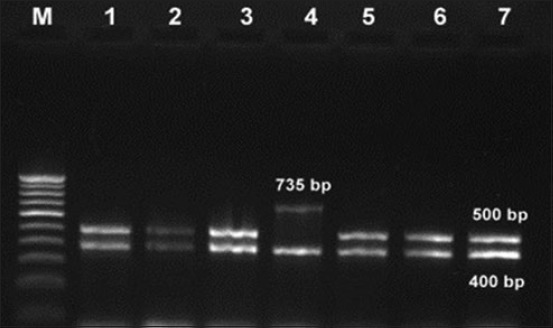
Multiplex polymerase chain reaction for *Campylobacter* genus and species identification. Lane M: 100 bp ladder, Lanes 1, 2, 3, 5, 6, 7: *Campylobacter coli*, Lane 4: *Campylobacter jejuni*.

On performing the multiplex PCR, all the 38 isolates amplified *cadF* gene and produced 400 bp amplicon suggesting the isolates belonging to genus *Campylobacter*. *C. coli* and *C. jejuni* species targeting *asp* gene (500 bp) and *hipO* gene (735 bp), respectively, were amplified in 29 (n=38) and 9 (n=38) isolates revealing a prevalence of 76.32% and 23.68%, respectively (Figure-2).

### Prevalence of *Campylobacters* among various sources

Of 609 samples screened, 38 were found positive for *Campylobacter* spp. with a prevalence of 6.24%. Of a total of 38 thermophilic *Campylobacters* detected, 9 (23.68%) were identified as *C. jejuni* and 29 (76.32%) as *C. coli*. Highest isolation was recorded from poultry ceca (15.52%), followed by poultry droppings (5.91%), cattle feces (4.92%), human stools (3.40%), and pig feces (2.82%). All sheep, goat, and laboratory animal fecal samples tested were negative for *Campylobacte*r spp. (Table-1).

**Table-1:** Distribution of *Campylobacter* isolates across various samples.

Sample source	Number of samples collected	Number of positive samples (%)	*Campylobacter* *jejuni* (%)	*Campylobacter* *coli* (%)
Poultry droppings	203	12 (5.91)	3 (25)	9 (75)
Poultry ceca	116	18 (15.52)	2 (11.11)	16 (88.89)
Cattle feces	61	3 (4.92)	1 (33.33)	2 (66.67)
Sheep and goat feces	36	0 (0)	0 (0)	0 (0)
Pig feces	71	2 (2.82)	0 (0)	2 (100)
Laboratory animals’ feces	34	0 (0)	0 (0)	0 (0)
Human stools	88	3 (3.41)	3 (100)	0 (0)
Total	609	38 (6.24)	9 (23.68)	29 (76.32)

### Virulence typing

**Figure-3:**
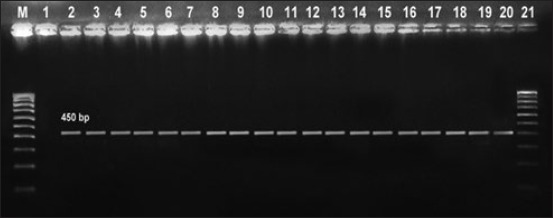
Gel electrophoresis of polymerase chain reaction (PCR) product *fla*A (450 bp), Lane M: 100 bp ladder, Lane 1: *Escherichia coli*, Lanes 2-20: *fla*A PCR product.

**Figure-4:**
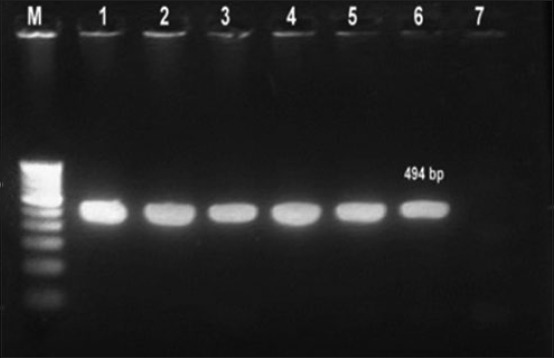
Gel electrophoresis of polymerase chain reaction (PCR) product *vir*B11 (494 bp), Lane M: 100 bp ladder, Lane 7: *Escherichia coli*, Lanes 1-6: *vir*B11 PCR product.

**Figure-5:**
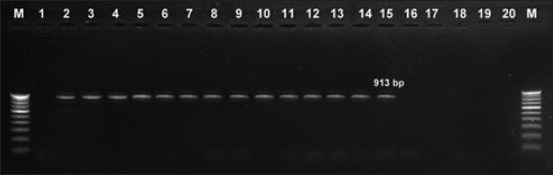
Gel electrophoresis of polymerase chain reaction (PCR) product *pld*A (913 bp), Lane M: 100 bp ladder, Lane 1 - Escherichia *coli*, Lanes 2-15: *pld*A PCR product.

Virulence typing was performed using four genes as targets. The results suggested that *cad*F, *vir*B11, and *pld*A genes were present in 38 (100%), 7 (18.42%), and 14 (36.84%) isolates, respectively (Figures-3-5). The second highest prevalence was found of *fla*A (flagellar motility) gene that was amplified in 37(97.37%) isolates. A total of 7 isolates (18.42%), 5 of *C. jejuni* and 2 of *C. coli*, were found to express all the four virulent genes (*cad*F, *fla*A, *vir*B11, and *pld*A) as shown in Tables-2 and 3.

**Table-2:** Prevalence of virulent genes in *Campylobacter* isolates recovered from various sources.

Source	Number of isolates	Virulence genes detected in *Campylobacter* spp.			

		*cad*F (%)	*fla*A (%)	*vir*B11 (%)	*pld*A (%)
Poultry droppings	12	12 (100)	12 (100)	2 (16.67)	5 (41.67)
Poultry ceca	18	18 (100)	18 (100)	3 (16.67)	4 (22.22)
Cattle feces	3	3 (100)	2 (66.67)	1 (33.33)	2 (66.67)
Sheep and goat feces	0	0 (0)	0 (0)	0 (0)	0 (0)
Pig feces	2	2 (100)	2 (100)	0 (0)	0 (0)
Laboratory animals’ feces	0	0 (0)	0 (0)	0 (0)	0 (0)
Human stools	3	3 (100)	3 (100)	1 (33.33)	3 (100)
Total	38	38 (100)	37 (97.37)	7 (18.42)	14 (36.84)

**Table-3:** Distribution of virulent genes among the *Campylobacter* isolates.

Source	*Campylobacter* spp.	Total number of isolates	Virulent genes detected in *Campylobacter* spp.			

			*cad*F	*fla*A	*vir*B11	*pld*A
Poultry droppings	*C. jejuni*	3	3	3	2	2
	*C. coli*	9	9	9	0	3
Poultry ceca	*C. jejuni*	2	2	2	1	1
	*C. coli*	16	16	16	2	3
Cattle feces	*C. jejuni*	1	1	1	1	1
	*C. coli*	2	2	1	0	1
Sheep and goat feces	*C. jejuni*	0	0	0	0	0
	*C. coli*	0	0	0	0	0
Pig feces	*C. jejuni*	0	0	0	0	0
	*C. coli*	2	2	2	0	0
Laboratory animals’ feces	*C. jejuni*	0	0	0	0	0
	*C. coli*	0	0	0	0	0
Human stools	*C. jejuni*	3	3	3	1	3
	*C. coli*	0	0	0	0	0

*C. jejuni*=*Campylobacter jejuni*, *C. coli*=*Campylobacter coli*

### AMR

Antibiotic resistance profile *Campylobacter* isolates were determined using eight antibiotics according to the CLSI 2015. Comparison of antibiotic susceptibilities of the isolates from different sources is shown in Tables-4 and 5. All isolates (100%, n=38) were resistant to NA, while all were sensitive to ERY and COT. Only 2 *(*5.3%) *C. coli* isolates were resistant to AMP and 1 *(*2.6%) *C. jejuni* was resistant to GEN. Of all the isolates, 13.16%, 18.42%, 23.68%, 36.84%, and 23.68% were intermediately resistant to AMP, GEN, LE, CIP, and CTR, respectively, probably reflecting a shift toward resistance.

**Table-4:** Antibiotic sensitivity pattern of the obtained thermophilic *Campylobacter* isolates.

Antibiotic	Total isolates	Number resistant	Number intermediate	Number sensitive	% of resistant isolates
AMP	38	2	5	31	5.3
GEN	38	1	7	30	2.6
ERY	38	0	0	38	0.0
LE	38	0	9	29	0.0
CIP	38	0	14	24	0.0
NA	38	38	0	0	100.0
CTR	38	0	9	29	0.0
COT	38	0	0	38	0.0

AMP=Ampicillin, GEN=Gentamicin, ERY=Erythromycin, LE=Levofloxacin, CIP=Ciprofloxacin, NA=Nalidixic acid, CTR=Ceftriaxone, COT=Co-trimoxazole

**Table-5:** Antibiotic sensitivity pattern among *Campylobacter* isolates.

Antibiotics	*Campylobacter* spp.	Number of isolates		

		Resistant	Intermediaste	Sensitive
Ampicillin	*C. jejuni*	0	1	8
	*C. coli*	2	4	23
Gentamicin	*C. jejuni*	1	1	7
	*C. coli*	0	6	23
Erythromycin	*C. jejuni*	0	0	9
	*C. coli*	0	0	29
Levofloxacin	*C. jejuni*	0	1	8
	*C. coli*	0	8	21
Ciprofloxacin	*C. jejuni*	0	3	6
	*C. coli*	0	11	18
Nalidixic acid	*C. jejuni*	9	0	0
	*C. coli*	29	0	0
Ceftriaxone	*C. jejuni*	0	2	7
	*C. coli*	0	7	22
Co-trimoxazole	*C. jejuni*	0	0	9
	*C. coli*	0	0	29

*C. jejuni*=*Campylobacter jejuni*, *C. coli=Campylobacter coli*

## Discussion

*Campylobacter* is one of the leading causes of gastrointestinal illnesses worldwide. The present study was done to determine the prevalence of thermophilic *Campylobacters* (*C. jejuni* and *C. coli*), their presence of virulence genes, and the antimicrobial susceptibility of the obtained isolates. Of 609 samples screened, 38 were detected to be positive for *Campylobacter* spp. showing the overall prevalence rate to be 6.24%. The findings were in accordance with the work of Rajagunalan [17] and Pandey [18] who accounted 6.9% and 5.34% prevalence rate of *Campylobacter* spp. in the same area, respectively. However, there are reports of a bit higher prevalence in the same study region 16% [19], 11.66% [20], and 13.54% [21], and the reason probably could be the variation in species of host, time and season of sample collection, and the difference in the sample source of various species. This study also showed that *C. coli* (29/38, 76.32%) was more prevalent than C*. jejuni* (9/38, 23.68%) isolates which was incorcondance with Rajagunalan [17] and Kumar [20]. In contrast to these findings, many workers including Rajendran *et al*. [22] and Deckert *et al*. [23] have reported the higher presence of *C. jejuni* than *C. coli*. The variation in the prevalence of *C. jejuni* and *C. coli* could be due to difference in the samples, animal species screened, and the geographical location of the study. The use of growth promoters and increased AMR in *C. coli* has also been reasoned for the variation [24].

Among different sample sources, the highest isolation rate was observed from poultry ceca (15.52%), followed by poultry droppings (5.91%), cattle feces (4.92%), human stools (3.40%), and pig feces (2.82%). The observations were in agreement with the findings of Humphrey *et al*. [25], who observed that, other than poultry digestive tract, digestive tracts of cattle, pigs, and human beings also act as the significant reservoir for *Campylobacter* species. The isolation rate in poultry ceca was higher (15.52%) than poultry droppings (5.91%). The difference could be because organisms undergo stress after excretion which may affect its survival and recovery rate. Moreover, keeping period of a bird in a flock enhances the colonization of *Campylobacters*. The collection of fecal and cecal samples from the same bird might give a clear picture of the prevalence of the organism.

None of the isolates were recovered from sheep and goat feces. Low prevalence of *Campylobacters* in sheep and goat has been reported by Salihu *et al*. [26], Zweifel *et al*. [27], and Cortes *et al*. [28] who reported a very low prevalence of *Campylobacter* spp. in sheep and goats as compared to other animals. As a contradiction to this finding, Lazou *et al*. [29], Mpalang *et al*. [30], and Karikari *et al*. [31] have recorded high prevalence in sheep and goat. The fecal samples of laboratory animals also did not yield any isolate which was dissimilar to the findings of Jensen *et al*. [32] and Nkogwe *et al*. [33] who reported *C. jejuni* infection in rats. Our findings in case of sheep, goat, and laboratory animals differed probably due to a lower number of samples screened. Moreover, better husbandry practices [26] followed could have resulted in the absence of *Campylobacters*. The laboratory animals were kept in a strict hygienic environment separated from other animals in the medical as well as veterinary colleges which could be the reason for the absence of *Campylobacter* organism from these samples.

Virulence typing was performed using four genes as targets. Of all the 38 isolates, all the isolates (100%) showed the presence of *cadF* genes. The other three *fla*A, *vir*B11, and *pld*A genes were present in 37 (97.37%), 7 (18.42%), and 14 (36.84%) isolates, respectively.

The *cad*F and *fla*A genes responsible for the expression of adherence and colonization were highly detectable in all the isolates. The presence of *cad*F gene highly conserved in *Campylobacters* and regarded as genus-specific has been reported by Konkel *et al*. [34] and Wieczorek *et al*. [35]. This protein is important for full binding capacity of the *Campylobacters* to the host intestinal epithelial cells. Rozynek *et al*. [36] and Wieczorek and Osek [37] have also identified this virulence gene to be present in the feces of poultry, animal, and human isolates. The presence of *fla*A gene which determines flagellar motility responsible for motility and colonization of enterocytes is one of the best-studied virulence markers and in this study was present in 37 (97.37%) isolates. Previous studies have also indicated that the detection rate of *fla*A gene is high (>95%).

The *vir*B11 and the *pld*A (phospholipase A) genes are responsible for invasion and survival within the host cells. The *vir*B11 gene was rarest among all the studied genes and was present in the isolates obtained from poultry droppings (16.67%), poultry ceca (16.67%), cattle feces (33.33%), and human stools (33.33%). Bang *et al*. [38] also found only 7.5% of the isolates from pigs and cattle to be positive for *vir*B11. Studies conducted on poultry, animals, and humans by Biswas *et al*. [15], Talukder *et al*. [39], Koolman *et al*. [40], and Jribi *et al*. [41] also, in fact, did not find the presence of *vir*B11 gene. The *pld*A gene was also detected in 36.84% isolates. Melo *et al*. [42] also detected the presence of *pld*A gene in 63.65% of the *C. jejuni* strains isolated from chicken meat. The presence of these genes poses a potential hazard to human health.

Antibiotic susceptibility profile of all the 38 isolates (29 *C. coli* and 9 *C. jejuni*) revealed 100% resistance to NA. Only 2 (5.3%) *C. coli* and 1 (2.6%) *C. jejuni* were resistant to AMP and GEN, respectively. Of all the isolates, 13.16%, 18.42%, 23.68%, 36.84%, and 23.68% were intermediately sensitive to AMP, GEN, LE, CIP, and CTR, respectively, probably reflecting a shift toward resistance. The variation in the antimicrobial sensitivity pattern of the *Campylobacter* isolates was noticed among various reports. A significant increase in the resistance for NA (46.7% of the isolates) and CIP (52.2%) was also observed in Spain [43]. However, in our study, all the isolates were found to be resistant to NA but sensitive to CIP and LE. Furthermore, 100% resistance to cephalothin and COT and 100% sensitivity to AMP, GEN, and ERY were reported [17]. Only 71.4% of isolates had sensitivity against NA. 80% and 77% of *Campylobacter* isolates obtained from Thailand and India, respectively, were found to be resistant against fluoroquinolones [44]. Higher resistance rates to CIP (95.8-99%, 85.4%, and 91%) have been reported in China [45], the United Arab Emirates [46], and South Africa [47], respectively. The development of resistance in the poultry and animals of the study area is a potential threat to human health. There is much fear that this resistance may spread to environment [48] which may further lead to difficult to treat cases.

## Conclusion

The present study highlights the existence of thermophilic *Campylobacters* in poultry, animals, and even in human samples. The recovery of positive isolates from humans emphasizes human-animal proximity that must have led to the transmission. The presence of virulence genes in the isolates marks their role in the establishment of the disease and thus modulates the clinical presentation in the host. Increasing antibiotic resistance against quinolone reflects the misuse/overuse of the antibiotics in the area.

## Authors’ Contributions

NR collected the samples and analyzed the samples. M designed the study. DK performed analysis of the data and AKU provided help as and when required and edited the manuscript. All authors have read and approved the final manuscript.
